# Synchronous Jejunal Sarcomatoid Carcinoma and Incidentally Associated Localized Peritoneal Malignant Mesothelioma

**DOI:** 10.7759/cureus.26270

**Published:** 2022-06-24

**Authors:** Mitsuhiro Tachibana, Masashi Nozawa, Kazuyasu Kamimura, Yutaka Tsutsumi

**Affiliations:** 1 Department of Diagnostic Pathology, Shimada General Medical Center, Shimada, JPN; 2 Department of Surgery, Shimada General Medical Center, Shimada, JPN; 3 Diagnostic Pathology Clinic, Pathos Tsutsumi (Tsutsumi Byori Shindanka Clinic), Inazawa, JPN

**Keywords:** p16/cdkn2a, asbestos, programmed death-ligand 1, tumor-infiltrating lymphocytes, localized malignant mesothelioma, sarcomatoid carcinoma, jejunal carcinoma

## Abstract

Sarcomatoid carcinoma (SCA) of the small bowel is a rare aggressive variant of small intestinal cancer accompanying a poor prognosis. The tumor primarily affects middle-aged and elderly patients. We report herein a 67-year-old Japanese male who manifested anemia. He had a history of asbestos exposure 30 years earlier. An abdominal computed tomography (CT) scan showed a 6.5-cm aneurysmal, dilated mass of the small intestine. Capsule endoscopy revealed a large circumferential hemorrhagic ulcerative lesion in the jejunum. Biopsy indicated sarcomatoid carcinoma, and partial resection of the small bowel and adjacent transverse colon and omentum was performed. In addition to the T3N0M0 jejunal giant sarcomatoid carcinoma (SCA), a 3-mm small localized peritoneal (omental) malignant mesothelioma (LMM) was also incidentally included. Synchronous presentation of small intestinal and mesothelial malignancies is extremely rare, and the avoidance of incorrect clinical staging is critically important. Surgical resection is still considered the best first-line therapy, because of a poor response to chemotherapy and radiotherapy. Dual-color fluorescent in situ hybridization (FISH) for p16/CDKN2A and chromosome 9 indicated homologous deletion of p16/CDKN2A in SCA and a normal pattern in LMM. Methylthioadenosine phosphorylase (MTAP) was negative in SCA but positive in LMM. Both tumors consistently expressed BRCA1-associated protein 1 (BAP1). Tumor necrosis factor receptor-associated factor 7 (TRAF7) was suppressed, and neural cell adhesion molecule L1 precursor (NCAML1/L1CAM) was agitated in both tumors. Diffuse and strong expression of programmed death-ligand 1 (PD-L1) and the association of tumor-infiltrating lymphocytes in SCA may indicate a potential for PD-L1-targeted immunotherapy for treating this type of aggressive cancer. PD-L1 was focally expressed in LMM. The postoperative course was uneventful for two years.

## Introduction

Primary small intestinal carcinoma is rare with an incidence of 0.5-0.8 per 100,000 population per year [1,2]. Sarcomatoid carcinoma (SCA) of the small intestine is an extremely rare, aggressive variant of small intestinal malignancy accompanying a poor prognosis [1-3]. The tumor primarily affects middle-aged and elderly patients with a mean age of 57 years at the time of presentation [3,4]. The cancer cells of this subtype display a mimicry of mesenchymal spindled cells. A variety of terms have been used for describing this tumor, including carcinosarcoma, pleomorphic carcinoma, and anaplastic giant cell carcinoma. Based on its ultrastructural and immunohistochemical characteristics, SCA became the most universally accepted term and is recommended for routine use in diagnostic surgical reports [4]. So far, only 32 cases of primary SCA of the small bowel have been reported in the English literature [3,4].

Localized malignant mesothelioma (LMM) of the peritoneum is also an infrequently encountered neoplasm that occurs as a discrete, solitary, and circumscribed nodule. LMM has been reported equally in both sexes, and patients have an age range from the 40s to the 70s. The tumors are sessile or pedunculated and range in size from a few to 10 cm [5].

We report herein a case of jejunal SCA and unexpected concurrent primary peritoneal LMM of small size, describing detailed clinical, histopathological, ultrastructural, immunohistochemical, and molecular features of the unusual malignancy coexistence.

## Case presentation

**Figure 1:**
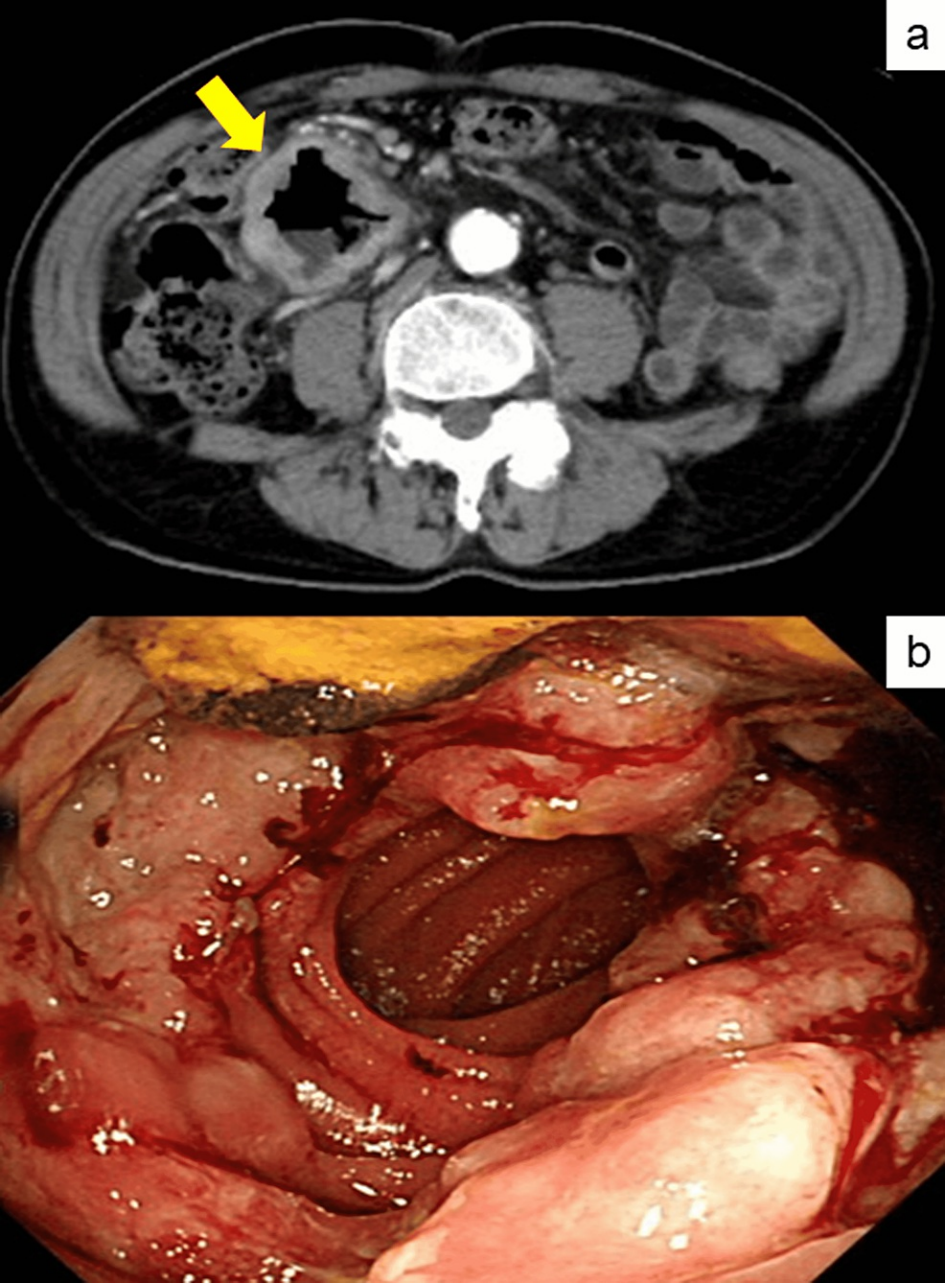
Clinical imaging. (a) CT scan showed a 6.5-cm aneurismally dilated mass in the small bowel (yellow arrow). (b) Capsule endoscopy revealed a large circumferential hemorrhagic mass in the jejunum.

A 67-year-old Japanese male was admitted to Shimada General Medical Center, Shimada, Shizuoka, Japan, with complaints of shortness of breath on exertion, palpitation, facial pallor, asitia, and decreased body weight. He had a history of asbestos exposure in plumbing work 30 years earlier. On physical examination, no mass was palpable on the abdomen. Laboratory data showed hypochromic microcytic anemia: hemoglobin, 7.4 g/dL; MCV, 70.8 fl; MCH, 20.8 pg; and MCHC, 29.4%. Albumin, serum iron, and ferritin levels were 3.1 g/dL, 10 g/dL, and 27.6 ng/mL, respectively. The serum levels of carcinoembryonic antigen (CEA) and carbohydrate antigen 19-9 (CA19-9) were within normal ranges (CEA, 1.6 ng/mL; CA19-9, 17.6 U/mL), while soluble interleukin-2 receptor was slightly elevated to 824 U/mL. An abdominal computed tomography (CT) scan showed a 6.5-cm aneurismally dilated mass (Figure 1a). Capsule endoscopy revealed a large circumferential hemorrhagic ulcerated mass in the jejunum at 20 cm from the ligament of Treitz, consistent with a primary malignancy, suspicious of malignant lymphoma (Figure 1b). Endoscopic biopsy of the mass revealed sarcomatoid carcinoma.

Partial resection of the small bowel and adjacent transverse colon and omentum was performed through a transabdominal incision. No ascites was noted at laparotomy. The postoperative course was uneventful for two years. No ascites retention was recorded during his illness. Neither chemotherapy nor radiotherapy was given.

Pathological findings

Gross Morphology

**Figure 2:**
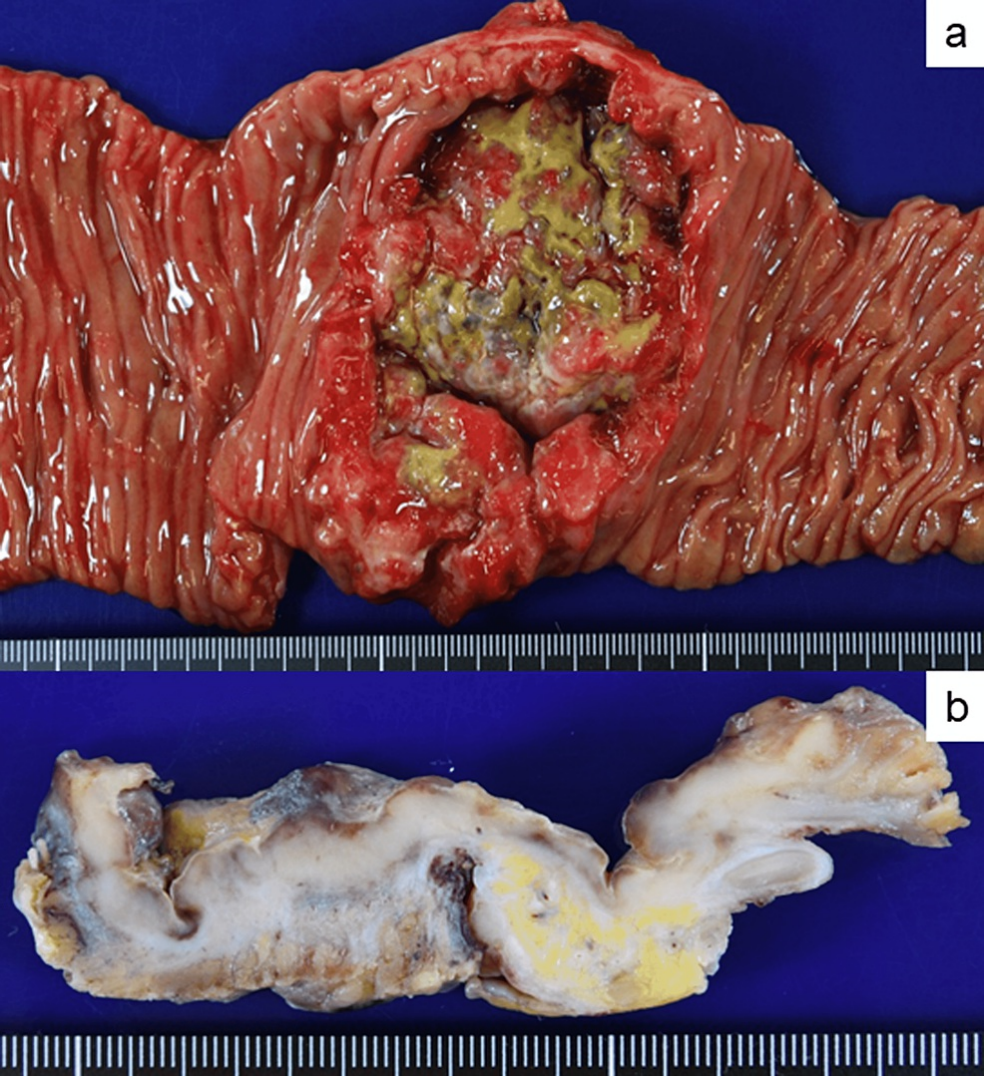
Macroscopic appearance of the resected jejunum. (a) The jejunal lesion exhibits an endophytic mass with deep central ulceration. (b) Cut surface demonstrates a transmurally invasive tumor with necrotic, hemorrhagic, and a fish flesh appearance.

The partial resection specimen contained a segment of the jejunum and transverse colon. The jejunal wall presented a red to yellowish endophytic circumferential ulcerated mass measuring 65 × 55 mm (Figure 2a).

On cross-section, the mass of the jejunum was necrotic, hemorrhagic, and white-colored with a fish flesh appearance (Figure 2b). The mass extended to the subserosal layer. Unexpectedly, a small peritoneal nodule, measuring 3 mm, was noted in the omentum. The second nodular lesion was solid, hard, and white-colored.

Microscopic findings

**Figure 3:**
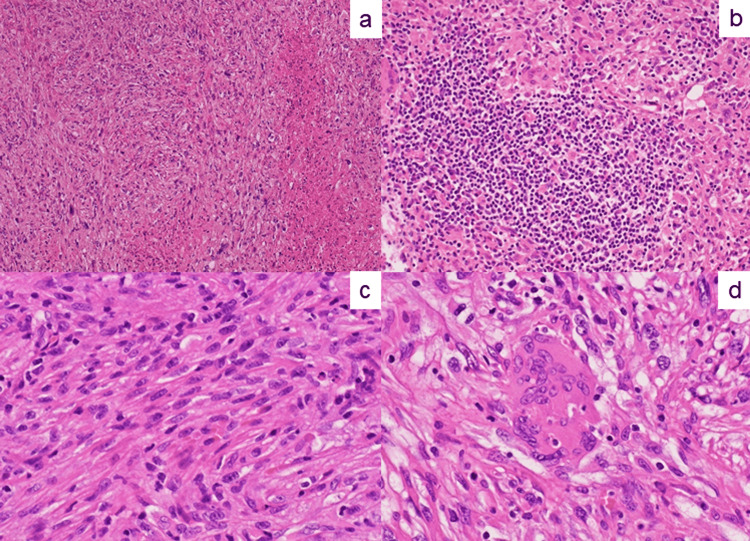
Microscopic findings of the jejunal tumor. (a) The sarcomatoid neoplasm consisting of spindled tumor cells with an irregular fascicular arrangement and focal coagulation necrosis (H&E); (b) tumor-infiltrating lymphocytes in the stroma (H&E); (c) neoplastic spindled cells accompanying large, elongated, and “cigar-shaped” nuclei (H&E); and (d) osteoclast-like giant cells with lymphocytic reactions (H&E).

Microscopically, the tumor of the jejunum diffusely grew with a poorly cohesive, sarcomatoid spindle-celled pattern in hematoxylin and eosin (H&E) preparations. Coagulation necrosis was frequently noted (Figure 3a). Tumor-infiltrating lymphocytes were associated with the stroma (Figure 3b). No glandular differentiation was recognized, nor was mucin formation. The spindled or polygonal tumor cells possessed large, round to elongated or “cigar-shaped” nuclei with irregular nuclear rims, condensed chromatin, and prominent nucleoli (Figure 3c). Bizarre nuclei were scattered. The rhabdoid appearance was associated (see also Figure 7a). There was brisk mitotic activity. Multinucleated giant cells were also noted, together with lymphocytic reactions (Figure 3d). No lymphovascular invasion was found. The tumor had invaded the subserosal adipose tissue. The surgical margins were free of tumors. In 13 regional lymph nodes examined, no metastatic carcinoma was identified. The Unio Internationalis Contra Control (UICC) stage of this invasive carcinoma was T3N0M0.

**Figure 4:**
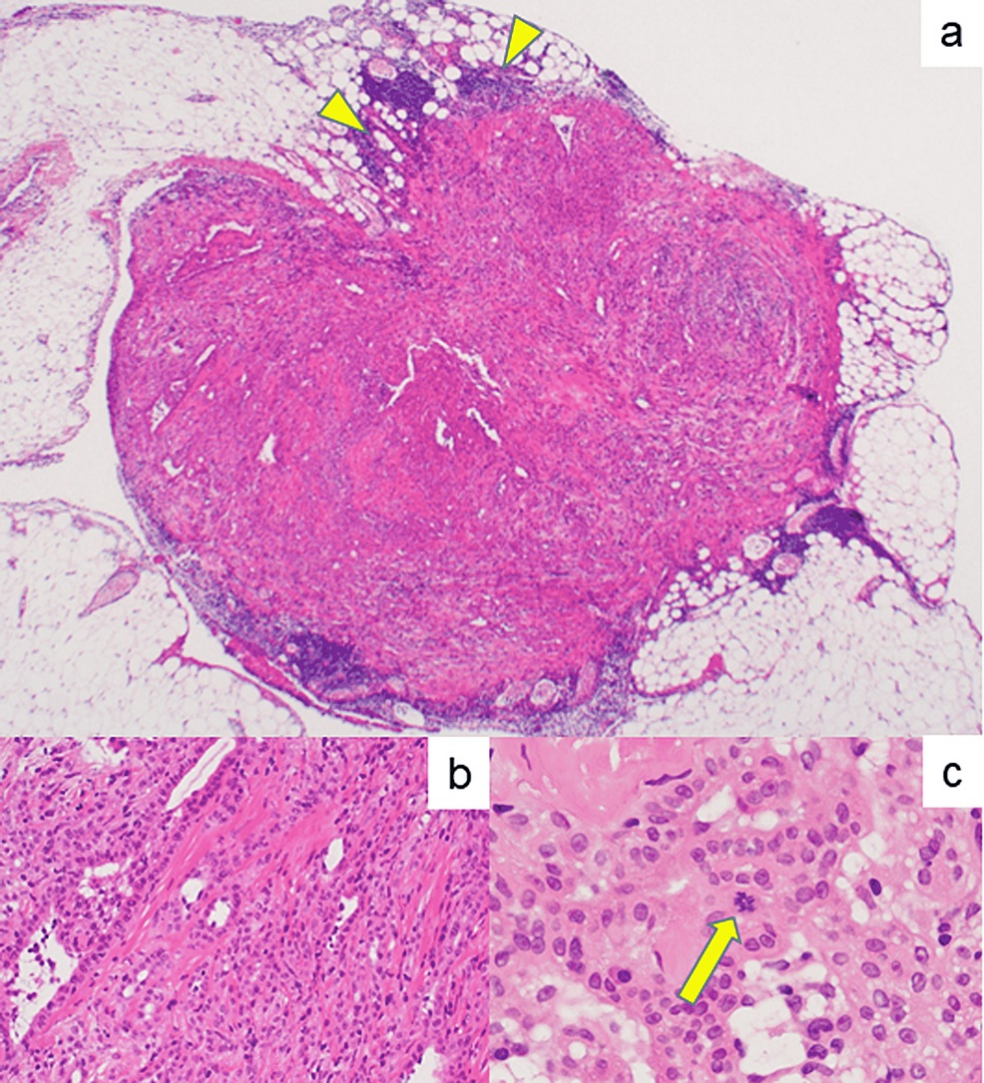
Microscopic findings of the small omental nodule measuring 3 mm. (a) An entire image of LMM with a loupe, with arrowheads showing invasion into the fat tissue (H&E). (b) Tumor cells showing tubular formation (H&E). (c) High-powered view showing mitotic activity in atypical mesothelium-like cells (arrow, H&E).

The peritoneal small nodule on the omentum, showing focal invasion into the fat tissue (Figure 4a), was composed of tubular structures with a histologic pattern compatible with malignant mesothelioma, epithelial type (Figure 4b, see also Figure 8a). The tubules were lined by mesothelium-like malignant cells with vesicular nuclei, prominent nucleoli, and relatively abundant cytoplasm. Mitotic activity was occasionally observed (Figure 4c). No lymphoid stroma was observed. The diagnosis of LMM was confirmed by Prof. Tohru Tsujimura, Division of Molecular Pathology, Department of Pathology, Hyogo College of Medicine, Nishinomiya, Hyogo, Japan, a known specialist in mesothelioma pathology.

Immunohistochemistry

**Figure 5:**
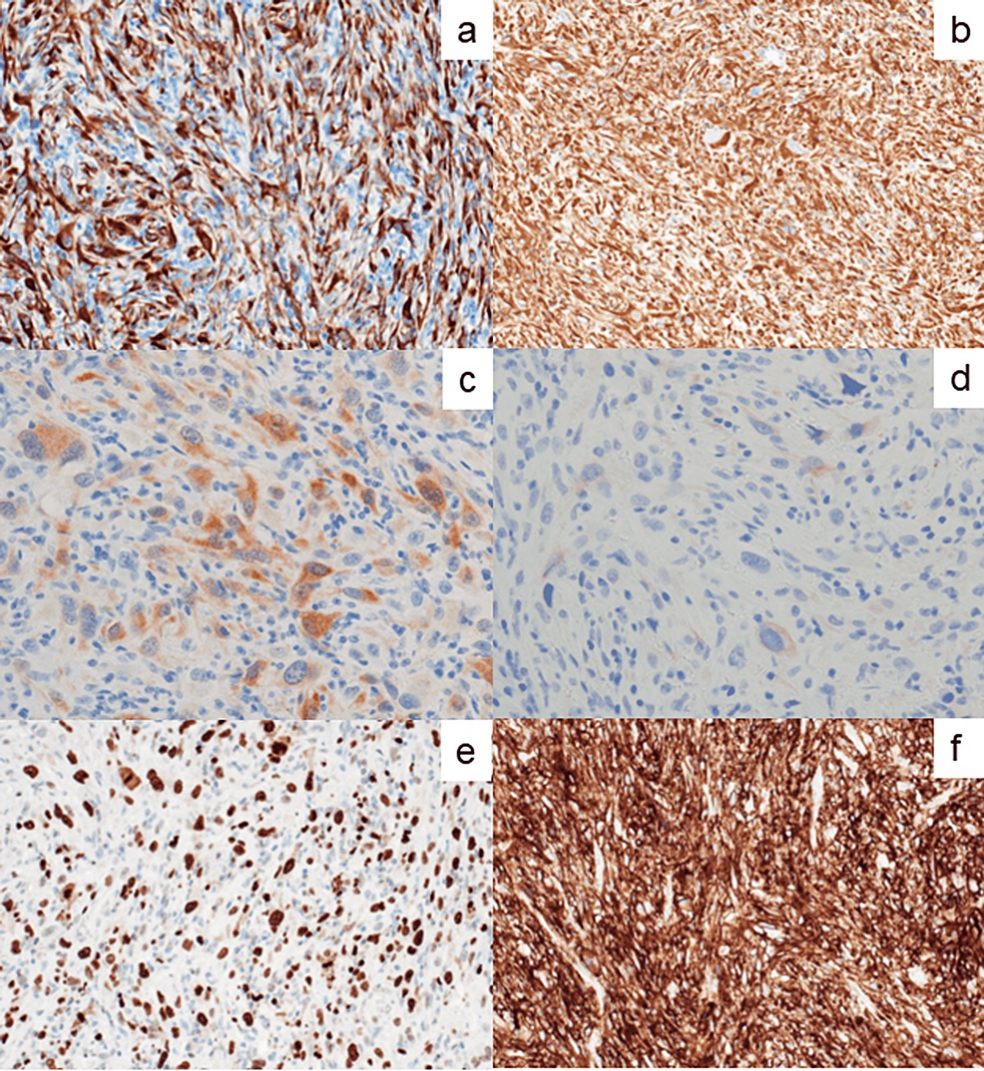
Immunohistochemical findings of the jejunal tumor. (a) CK7, (b) vimentin, (c) calretinin, (d) mesothelin, (e) Ki-67, and (f) PD-L1. The sarcomatoid cancer cells diffusely express CK7 and vimentin. Focal expression of mesothelial cell markers (calretinin and mesothelin) is observed, the Ki-67 labeling index is high, and the spindled tumor cells diffusely and strongly express PD-L1.

As indicated in Figure 5, the spindled jejunal cancer cells revealed diffuse immunoreactivity for vimentin, cytokeratin (CK)-AE1/AE3, CK-CAM5.2, CK7, and programmed death-ligand 1 (PD-L1, clone: 22C3). The Ki-67 labeling index was as high as 80%. PD-L1 was diffusely and strongly expressed in 100% tumor cells. A small number of the atypical sarcomatoid cells were immunoreactive for mesothelial cell markers such as calretinin, mesothelin, Wilms tumor-1 (WT-1), and sialylated protein HEG homolog 1 (HEG1). A small percentage of the atypical cell nuclei were immunostained for p53, indicating a wild-type p53 expression pattern [6]. Negative markers included CK5/6, CK10, CK13, CK17, CK20, CK-34bE12, claudin 4, CEA, CD34, CD117 (c-kit), S-100 protein, podoplanin (D2-40), alpha-smooth muscle actin (SMA), heavy caldesmon, desmin, discovered on GIST-1 (DOG1), melan A, human melanin black-45 (HMB45), chromogranin A, synaptophysin, neural cell adhesion molecule (NCAM = CD56), CD30, alpha-fetoprotein (AFP), human chorionic gonadotropin-beta (HCG-b), Sal-like protein 4 (SALL-4), glypican 3, and SRY-related HMG-box 10 (SOX10). Infiltration of CD45-positive/CD8-positive/granzyme B-focally positive small lymphocytes and CD68-positive macrophages was closely associated with the tumor growth. Multinucleated giant cells of CD68-positive macrophage lineage were also occasionally clustered.

**Figure 6:**
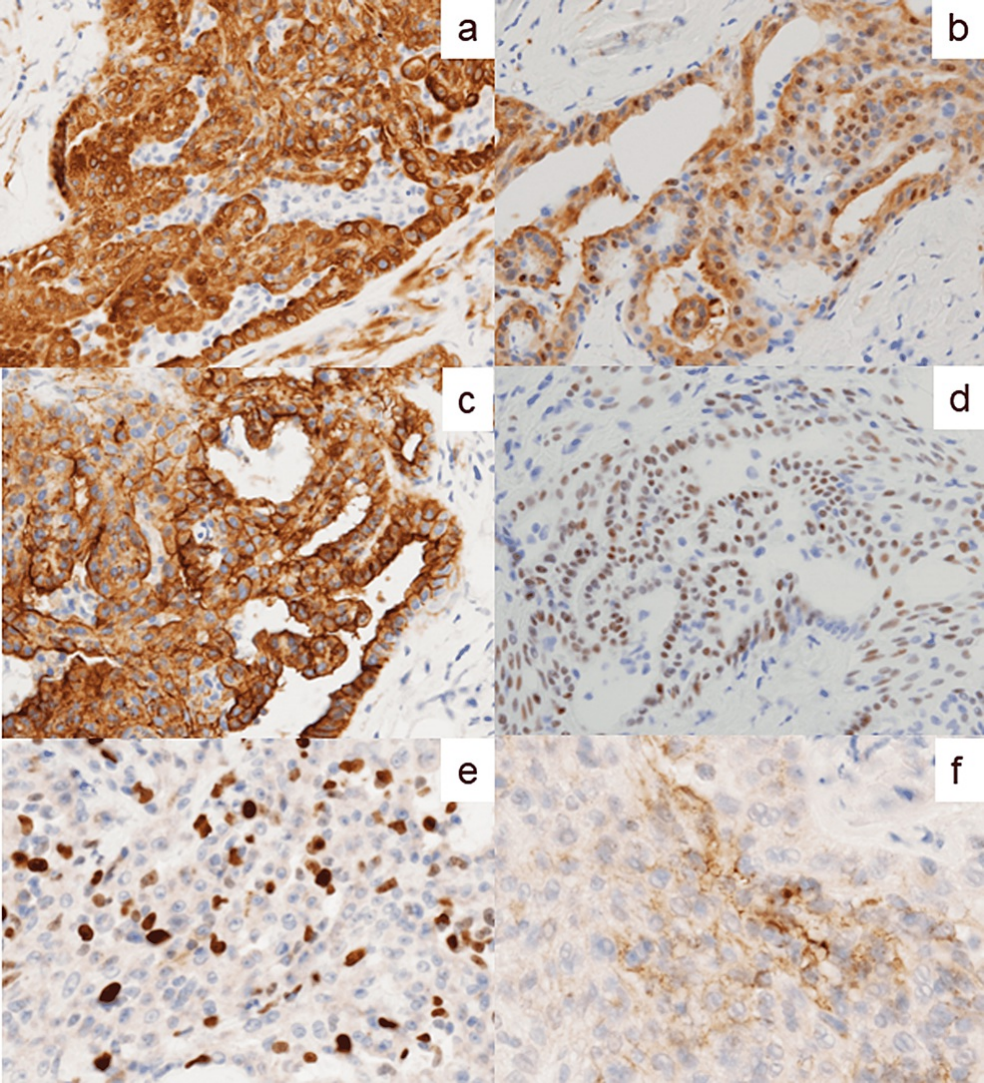
Immunohistochemical findings of the omental nodule. (a) CK-AE1/AE3, (b) calretinin, (c) mesothelin, (d) WT-1, (e) Ki-67, and (f) PD-L1. The LMM cells diffusely express CK-AE1/AE3, calretinin, mesothelin, and WT-1. The Ki-67 labeling index is around 20%. PD-L1 is focally immunoreactive.

As displayed in Figure 6, the small peritoneal nodule showed diffuse immunoreactivity for mesothelial cell markers such as calretinin, mesothelin, WT-1, podoplanin, and HEG1. CK7, CK-AE1/AE3, CK-CAM5.2, CK-34bE12, and vimentin were also diffusely expressed. CK20 was focally positive. p53 revealed a wild-type, focal expression pattern. The Ki-67 labeling index was around 20%. PD-L1 was expressed in 5% of the tumor cells. No staining was seen for CK5/6, CK10, CK13, CK17, claudin 4, CEA, CD34, CD117, S-100 protein, and SMA.

Additional molecular and immunohistochemical analysis for p16INK4a (p16) and related markers

**Figure 7:**
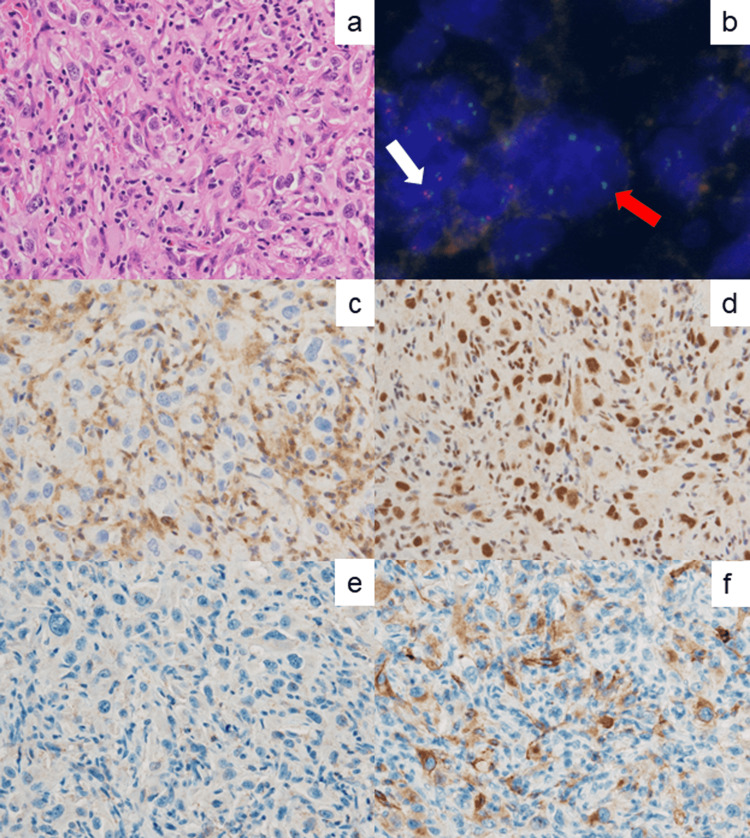
Molecular and immunohistochemical analysis for p16INK4a (p16) and related markers in the jejunal tumor. (a) H&E, (b) dual-color FISH for p16/CDKN2A (red) and chromosome 9 (green), (c) MTAP, (d) BAP1, (e) TRAF7, and (f) NCAML1/L1CAM. The sarcomatoid cancer cells often with a rhabdoid appearance show homologous deletion of p16 (red arrow). Inflammatory cells reveal a normal pattern (white arrow). MTAP is negative in the tumor cells, whereas BAP1 was expressed in the nuclei. The negativity of TRAF7 is closely related to the activated expression of NCAML1/L1CAM.

**Figure 8:**
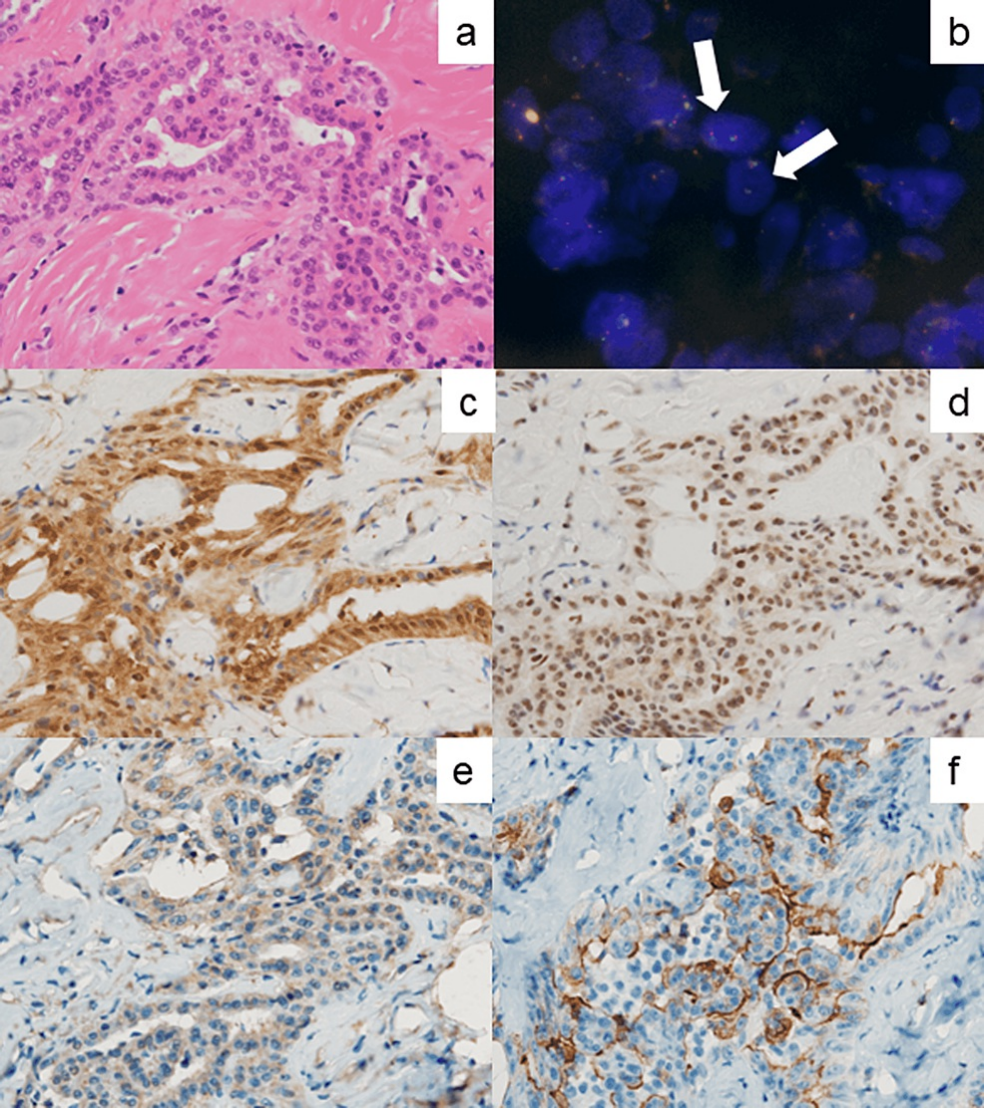
Molecular and immunohistochemical analysis for p16INK4a (p16) and related markers in the omental tumor. (a) H&E, (b) dual-color FISH for p16/CDKN2A (red) and chromosome 9 (green), (c) MTAP, (d) BAP1, (e) TRAF7, and (f) NCAML1/L1CAM. LMM revealed a normal pattern of p16. MTAP and BAP1 are diffusely expressed. TRAF7 expression is suppressed (only weakly and focally positive), and NCAML1/L1CAM is strongly expressed along the plasma membrane.

Fluorescent in situ hybridization (FISH) analysis using paraffin sections was performed for detecting p16/CDKN2A deletion. Dual-color FISH for p16/CDKN2A and chromosome 9 was employed [7,8]. Homologous deletion of p16 was detected in most atypical cells of SCA (84%; 109/129) (Figure 7b). In contrast, LMM revealed a normal pattern of p16 in most cells (94%; 101/107) (Figure 8b). Immunostaining for p16CDKN2A (p16) and CDKN2A (p14ARF) gave negative results in both tumors.

Then, additional immunohistochemical analysis was performed for p16INK4a (p16)-related markers [9]. The atypical cells of SCA were negative for methylthioadenosine phosphorylase (MTAP) and tumor necrosis factor receptor-associated factor 7 (TRAF7), whereas BRCA1-associated protein 1 (BAP1) and neural cell adhesion molecule L1 precursor (NCAML1/L1CAM) were expressed. The representative features are illustrated in Figure 7.

The LMM cells with normal p16 gene status diffusely expressed MTAP and BAP1. TRAF7 was weakly and focally positive, in association with the activated expression of NCAML1/L1CAM (Figure 8).

Ultrastructural findings

A paraffin block of the biopsy specimen was processed for ultrastructural observation, as reported previously [10,11]. The specimen was routinely fixed in phosphate-buffered 10% formalin, pH 7.4, and embedded in paraffin. The area with tumor cells was dug out of the paraffin block as 1-mm cubes. After deparaffinization overnight, the tissue block was dehydrated in graded series of alcohol, refixed at 4°C overnight in 2.5% glutaraldehyde buffered with 0.1 M sodium cacodylate at pH 7.4, osmified for two hours with sodium cacodylate-buffered 1% osmium tetroxide, embedded in epoxy resin (Epon 812), and polymerized overnight in a 70°C oven. Ultrathin sections were prepared with a diamond knife at 80 nm thickness and stained with uranyl acetate and lead citrate. Images were photographed on a JEOL JEM1400 Flash Electron microscope (Japan Electron Optics Laboratory, Akishima, Japan).

**Figure 9:**
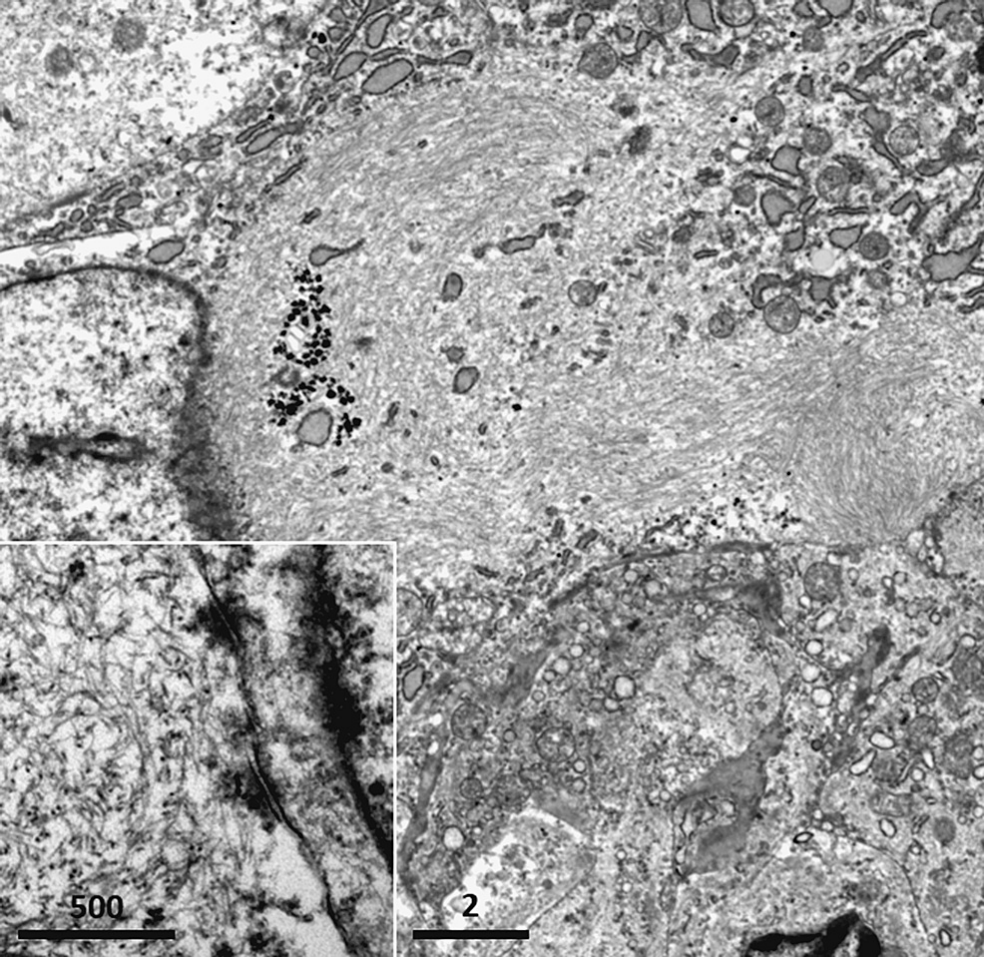
Electron microscopic appearance of the jejunal tumor. Bundles of intermediate filaments aggregated to form rhabdoid-like cells (bar = 2 mm). Inset: Desmosomal attachment is infrequently observed (bar = 500 nm). Fine morphological preservation is satisfactory enough.

The neoplastic cells of the main tumor (SCA) possessed oval or indented nuclei with peripherally aggregated chromatin. The cytoplasm was plump, and lysosomal granules or vesicles were clustered. Bundles of intermediate filaments aggregated to form rhabdoid-like cells. Microvillous structures were focally identified. Junctional complexes were poorly developed: tight junctions and desmosomes were seen only focally. The representative ultrastructural features are demonstrated in Figure 9.

## Discussion

Sarcomatoid carcinoma (SCA) is an extremely rare type of small intestinal carcinoma displaying both carcinomatous and sarcomatous features [12]. The tumor was first described by Dikman and Toker in 1973 [13], who named it as enteroblastoma. Gastrointestinal sarcomatoid tumors occur relatively frequently in the stomach, gallbladder, and esophagus [1]. Andrawes et al. [3] reviewed SCA of the small intestine: the jejunum was the most common site, followed by the ileum, and only one case of duodenal SCA was recorded.

Small intestinal SCA commonly shows a polypoid or fungating appearance with or without ulceration and often with necrotic and hemorrhagic features [1,3]. The average size is 7.5 cm with a range of 3-16 cm [4]. Lymphovascular invasion is noted in 55% of the lesions [4]. Immunohistochemical positivity of cytokeratin proteins significantly helps the differential diagnosis, excluding the possibility of a gastrointestinal stromal tumor, leiomyosarcoma, epithelioid angiosarcoma, amelanotic melanoma, and malignant lymphoma [14]. Ultrastructural studies also contribute to the proof of the epithelial nature of SCA. In the present case, desmosomal attachment and microvillous surface structure were focally observed. It should be noteworthy that the use of routinely prepared paraffin block gave satisfactory fine structural preservation.

Surgical resection should be the first choice of therapy for SCA of the small intestine. Chemotherapy and radiotherapy are poorly effective. During a three-year follow-up study, 70% of the patients died within two months [15]. In the present case, no adjuvant therapy was given, but the patient is alive without recurrence or distant metastasis for two years after surgery.

Clinical trials injecting monoclonal antibodies against immune checkpoint programmed death protein 1 (PD-1) and its ligand, PD-L1, reported a promising antitumor activity in several malignancies. Upregulated PD-L1 expression on the tumor cells mediates immune evasion via the activation of the PD-1/PD-L1 pathway and the suppression of effector immune responses [16]. Interestingly, we observed diffuse and strong expression of PD-L1 in the primary SCA of the jejunum, indicating a potential for PD-L1-targeted immunotherapy for the treatment of this type of cancer. The association of tumor-infiltrating lymphocytes should be compatible with the expression of PD-L1 on the tumor cells [17].

Peritoneal malignant mesothelioma belongs to a rare tumor [18]. It was first described in 1908 by Miller and Wynn [19]. Similar to pleural mesotheliomas, peritoneal mesotheliomas reveal a close etiological relationship with asbestos exposure [20]. The present case has a history of asbestos exposure 30 years earlier. Allen et al. [21] reported 23 cases of LMM. Twenty-one LMMs were pleural, and two were peritoneal in origin. Sixteen tumors revealed a microscopic appearance of epithelial mesotheliomas, six had mixed epithelial and sarcomatous patterns, and one was purely sarcomatous. Unlike diffuse malignant mesotheliomas, LMMs do not spread and thus can be successfully excised without recurrence in a considerable percentage of the patients. In other cases, however, LMMs recur after surgery and may metastasize [5]. LMM of peritoneal origin in the present case was incidentally observed and was very small in size (3 mm). The microscopic invasive nature of the lesion excluded the possibility of reactive mesothelial hyperplasia.

Panels of immunohistochemical markers were valuable for distinguishing SCA of the small intestine from LMM. None of the markers are 100% specific or 100% sensitive; thus, the combination of immunohistochemical markers for carcinoma and mesothelioma should be employed. We chose such mesothelial markers as calretinin, mesothelin, WT-1, D2-40, and HEG1, all of which showed distinct positivity in LMM. Regarding intermediate filament panels, both jejunal and peritoneal tumors expressed vimentin, CK-AE1/AE3, CK-CAM5.2, and CK7. LMM was further positive for CK-34bE12 and CK20 but unexpectedly negative for CK5/6. PD-L1 was also focally (5%) positive in the mesothelioma cells.

The significance of limited (minor) expression of mesothelial markers (calretinin, mesothelin, WT-1, and HEG1) in SCA cells should be discussed briefly. It has been documented that adenocarcinomas of the breast, lung, and pancreas may express mesothelial markers, including calretinin [22-24]. Kenmotsu et al. [25] described the expression of mesothelial markers in a pleomorphic or sarcomatoid component of lung cancer. We must not forget that mesothelial markers are not 100% specific to mesotheliomas, particularly when the markers are expressed only focally as was so in the present case.

The p16/CDKN2A gene status and the expression of the related markers were additionally analyzed in both tumors. SCA showed homologous deletion of the p16 gene with the lack of p16 protein expression. The same gene deletion was observed in a variety of human malignancies, including lung cancer, melanoma, glioma, and leukemia [26]. Sarcomatoid tumors of the lung also accompanied p16 deletion [27]. The negativity of MTAP in SCA suggested the homologous deletion of the MTAP gene, which is located adjacent to the locus of the CDKN2A gene [26]. BAP1 was expressed in the nuclei of SCA, indicating an unremarkable BAP1 gene status without BAP1 loss. The lack of TRAF7 expression suggested the mutation of the TRAF7 gene that resulted in the activated expression of NCAML1/L1CAM, whose gene is located downstream of the TRAF7 gene [28].

In contrast, LMM revealed a normal pattern of the p16/CDKN2A gene. The LMM cells showed weak and focal expression of TRAF7 (suggesting a TRAF7 gene mutation) with subsequently activated expression of NCAML1/L1CAM. Diffuse expression of MTAP and BAP1 suggested a normal status of these genes. So far, the homologous deletion of the p16 gene has been identified in diffuse malignant mesothelioma [7-9]. Mutation of the BAP1 gene is known to induce diffuse malignant mesothelioma [29]. In well-differentiated papillary mesothelioma, the mutation of the TRAF7 gene provokes the overexpression of the L1CAM protein, while the p16 gene and the BAP1 gene show a normal status [28]. Offin et al. [30] described that peritoneal mesotheliomas were molecularly characterized by TRAF7 alterations rather than p16-related cell cycle alterations. The pattern in peritoneal LMM, epithelial type, in the present case resembled that in well-differentiated papillary mesothelioma.

Synchronous jejunal SCA and coexistent (incidental) primary peritoneal LMM are extremely rare. The differential diagnosis is important for preventing incorrect clinical staging. In addition to histopathological morphology, immunohistochemistry is critically effective for making a definitive diagnosis. In fact, the immunohistochemical evaluation effectively helped exclude the possibility of peritoneal dissemination of SCA in the present case. The present case also suggested a potential value of PD-L1 as an important clinical or prognostic biomarker in jejunal SCA.

## Conclusions

We described a surgical case with a rare combination of SCA of the jejunum and incidentally associated 3-mm LMM of the peritoneum. Detailed histopathological, immunohistochemical, and molecular analyses contributed not only to making definite pathological diagnoses but also to exactly assessing the clinical stage of the small bowel malignancy. The usefulness of routinely prepared paraffin blocks for ultrastructural evaluation should also be emphasized. Immunohistochemical identification of PD-L1 may be of value as a therapeutic biomarker for SCA accompanying lymphoid stroma.
